# National and Subnational Burden of Cardiovascular Diseases in Iran from 1990 to 2021: Results from Global Burden of Diseases 2021 study

**DOI:** 10.5334/gh.1429

**Published:** 2025-05-16

**Authors:** Mahsa Heidari-Foroozan, Melina Farshbafnadi, Ali Golestani, Sepehr Younesian, Hosein Jafary, Mohammad-Mahdi Rashidi, Ozra Tabatabaei-Malazy, Nazila Rezaei, Mostafa Moghimi Kheirabady, Arash Bagherian Ghotbi, Seyyed-Hadi Ghamari

**Affiliations:** 1 Non-Communicable Diseases Research Center, Endocrinology and Metabolism Population Science Institute, Tehran University of Medical Sciences, Tehran, Iran; 2 Student research center committee, School of Medicine, Shahid Beheshti University of Medical Sciences, Tehran, Iran; 3 School of Medicine, Tehran University of Medical Sciences, Tehran, Iran; 4 School of Medicine, Golestan University of Medical Sciences, Gorgan, Iran; 5 Endocrinology and Metabolism Research Center, Endocrinology and Metabolism Clinical Sciences Institute, Tehran University of Medical Sciences, Tehran, Iran; 6 Medical student, Non-Communicable Diseases Research Center, Endocrinology and Metabolism Population Science Institute, Tehran University of Medical Sciences, Tehran, Iran

**Keywords:** Cardiovascular Diseases, GBD, Epidemiology, Embolism, Iran, Myocardial Ischemia

## Abstract

**Introduction:**

In 2021, cardiovascular diseases (CVD) caused around 20.5 million deaths worldwide, making them a major health concern.

**Methods:**

Incidence, prevalence, death, years of life lost (YLL), years lived with disability (YLD), and disability-adjusted life years (DALYs) were the burden measures that were assessed. All measures are reported as both all-age numbers and age-standardized rates (ASR) with 95% uncertainty intervals (UI). Decomposition analysis was conducted on CVD incidence.

**Results:**

From 1990 to 2021, all-age CVD prevalence in Iran increased by 182.6% (2.9 to 8.3 million cases), with males consistently showing higher age-standardized prevalence rates (ASPR) than females (11,350 vs. 9,431 per 100,000 in 2021). ASPR remained stable nationally (9,956 to 10,386 per 100,000), peaking in adults ≥80 years. Incident cases rose by 159.6% (0.36 to 0.92 million), driven by population growth (49.5%) and aging (136.2%), while age-standardized incidence rates (ASIR) declined by 28.3% (1,337 to 1,197 per 100,000); with males (1,336) exhibiting higher rates than females (1,060) in 2021. All age deaths doubled (86,527 to 169,582) during this period, but age-standardized death rates (ASDR) decreased substantially by 42.97% (446 to 255 per 100,000). DALYs increased by 53.7% (2.4 to 3.7 million), though age-standardized DALY rates dropped 45.3% (9,096 to 4,977 per 100,000), dominated by ischemic heart disease (2,731 ASR) and stroke (1,229 ASR). High systolic blood pressure, dietary risks, and LDL cholesterol remained the leading contributors to DALYs nationwide.

**Conclusion:**

Iran’s rising CVD burden demands prioritizing cardiac care infrastructure in underserved provinces like Golestan, enforcing sodium reduction policies aligned with Iran’s existing trans-fat regulations, and integrating sex-specific programs such as tobacco control for males and community hypertension screening for women are critical. Multisectoral collaboration, including urban design promoting physical activity and subsidies for whole grains, must address provincial inequities exacerbated by Iran’s aging population and dietary risks.

## Introduction

The rapid expansion of the global economy and significant lifestyle shifts have driven a steady rise in the prevalence of chronic diseases, with cardiovascular disease (CVD) leading as the primary cause of death worldwide. In Iran, these global trends are mirrored by specific socio-economic and lifestyle changes that have significantly influenced the epidemiology of CVD (1). Encompassing a range of conditions affecting the heart and blood vessels—including ischemic heart disease (IHD), stroke, heart failure, hypertensive heart disease, and peripheral arterial disease—CVD represents a critical global health challenge. In 2021 alone, CVDs accounted for approximately 20.5 million deaths globally (2). This burden is projected to escalate, with CVD deaths expected to exceed 23 million annually by 2030 (3). In many high-income countries, despite the rising burden of CVD, age-standardized mortality rates have steadily declined in recent decades. This reduction can largely be attributed to effective primary and secondary preventive interventions, as well as continuous health promotion programs.

While age-standardized mortality rates have declined in many high-income countries due to advancements in prevention and health promotion, 80% of CVD-related deaths occur in low- and middle-income countries (LMICs) (4). These regions face disproportionate challenges, including limited healthcare resources and high economic burdens. Between 2011 and 2015, the economic impact of CVDs in LMICs was estimated at $3.7 trillion (5). Iran is no exception, where the average cost per patient reached $1,881.4 in 2016, culminating in total CVD costs of $1.16 billion that year (6).

Existing studies on CVD in Iran, while valuable, have been limited by narrow temporal or regional scopes(7,8), a focus on individual CVD subtypes (9), or incomplete reporting of burden metrics such as incidence, prevalence, and disability-adjusted life years (DALYs) (10). Few have systematically integrated risk factor analysis with disease burden trends, and none have employed decomposition methods to disentangle the drivers of rising CVD incidence (6).

In this study as a part of global burden of the disease (GBD) 2021 study, we address these gaps by presenting the most comprehensive analysis of CVD burden in Iran from 1990 to 2021, covering all subtypes—including ischemic heart disease, stroke, and aortic aneurysm—at national and provincial levels. We report trends in incidence, prevalence, mortality, and DALYs, while systematically linking these trends to modifiable risk factors. Crucially, we introduce decomposition analysis to quantify the contributions of population growth, aging, and incidence rate changes, offering novel insights into the drivers of rising CVD cases. This approach advances Iran’s CVD research by enabling policymakers to prioritize interventions addressing specific demographic or epidemiological factors.

## Methods

### Overview

This study utilized data from the GBD 2021 project, which includes health metrics such as incidence, prevalence, deaths, Years of Life Lost (YLLs), Years Lived with Disability (YLDs), and DALYs related to CVDs in Iran. The GBD 2021 data, compiled by the Institute for Health Metrics and Evaluation (IHME), provides the latest information on the annual burden of 371 diseases and injuries, 288 causes of deaths and 88 risk factors from 1990 to 2021 across 25 age groups in 204 countries and 811 sub-national locations, disaggregated by sex as well as combined (11,12,13). The GBD and this study adhere to the Guidelines for Accurate and Transparent Health Estimates Reporting (GATHER) in all steps of its analytical process and estimation methods (14). All data used in this study are publicly accessible via the GBD Compare (https://vizhub.healthdata.org/gbd-compare) and GBD Results (http://ghdx.healthdata.org/gbd-results-tool) web pages.

### Data sources

Systematic reviews, population surveys, and hospital administrative data were utilized, with network meta-analysis conducted using meta-regression—Bayesian, regularized, trimmed (MR-BRT) to adjust for study-level differences in case definition or measurement methods. Vital registration data were employed for all cardiovascular causes, while verbal autopsy data were used for total cardiovascular disease, ischemic heart disease, and total stroke, all mapped to the GBD cause list. For GBD 2021, all verbal autopsy data sources within the cardiovascular envelope underwent systematic review. Data sources displaying implausibly low or high values, which could lead to unrealistic time trends, were identified as outliers. Specific details on the excluding criteria for CVDs data source are documented elsewhere (12,13). All input sources and their metadata are accessible through the GBD 2021 Sources Tool (https://ghdx.healthdata.org/gbd-2021/sources)(Supplementary material 1).

### Definition

The GBD Study 2021 classifies causes into four hierarchical levels with CVDs categorized as Level 2 causes within the subset of non-communicable diseases (NCDs). CVDs are further divided into 12 subcategories including stroke, ischemic heart disease (IHD), atrial fibrillation/flutter, lower extremity peripheral artery disease, hypertensive heart disease, myocarditis, cardiomyopathy, endocarditis, pulmonary arterial hypertension, rheumatic heart disease, aortic aneurysm and other cardiovascular and circulatory diseases. Noteworthy that, under the GBD classification system, heart failure is not considered an independent cause within CVDs. Instead, it is classified as an impairment that contributes to the burden of several underlying causes, encompassing nearly all CVDs. The detailed definition of each cause along with their corresponding International Classification of Diseases (ICD) codes are listed elsewhere (12,13).

### Statistical analysis

DALYs were calculated as the sum of YLLs and YLDs, representing the comprehensive health burden of each cause. YLDs were estimated by multiplying disease prevalence by the corresponding disability weights, which were derived from surveys of the general population. YLLs, reflecting premature mortality, were computed by multiplying the estimated number of deaths by the standard life expectancy at the age of death. All summary measures were derived using 500 draws, with 95% uncertainty intervals calculated as the 2.5th and 97.5th percentiles of the posterior distribution of model draws. Additionally, the age-standardized rates were reported by standardizing the age distribution by the GBD standard global population age structure to address differences in population age distribution. This standard population is derived from the population structure of all national locations with populations exceeding 5 million people (12,13,15). To assess the association between SDI and burden measures in Iran, locally estimated scatterplot smoothing (LOESS) regression was performed, considering the SDI values of different provinces over time as the independent variable and the age-standardized rates of burden measures as the dependent variables.

#### Decomposition analysis

To assess the factors driving changes in the incidence of CVDs we employed a decomposition analysis using two distinct scenarios. In the first scenario, age-specific incidence rates from 1990 were applied solely to the 2021 population size, thereby estimating the expected number of cases under the assumption that the incidence rates remained unchanged over time. The difference between this estimate and the baseline (1990) case count quantifies the impact of population growth. In the second scenario, the same 1990 age-specific incidence rates were applied to the full 2021 population data—accounting for population size, age structure, and sex distribution—to generate an estimate that reflects both population growth and changes in demographic composition. The difference between the observed 2021 case count and this estimate represents the effect of changes in age-specific incidence rates over time. Finally, the difference between the estimates from the two scenarios was computed to isolate the contribution of population aging, that is, the impact of shifts in the age and sex distribution on the overall CVDs incidence. This analytical framework enables us to disentangle the relative contributions of population growth, changes in incidence rates, and population aging to the observed trends in CVDs incidence (16,17).

#### Sociodemographic index (SDI)

SDI is a composite measure that reflects the development level of countries or regions based on fertility rates among females under the age of 25. Higher income and education levels are fertility rates among females under the age of 25. Higher income and education levels are associated with better health outcomes, while higher fertility rates among young women typically correlate with poorer health outcomes (18).

#### Software

Data visualizations and analyses were performed using Python (version 3.10.13).

## Results

### Overall burden

**Figure 1:**
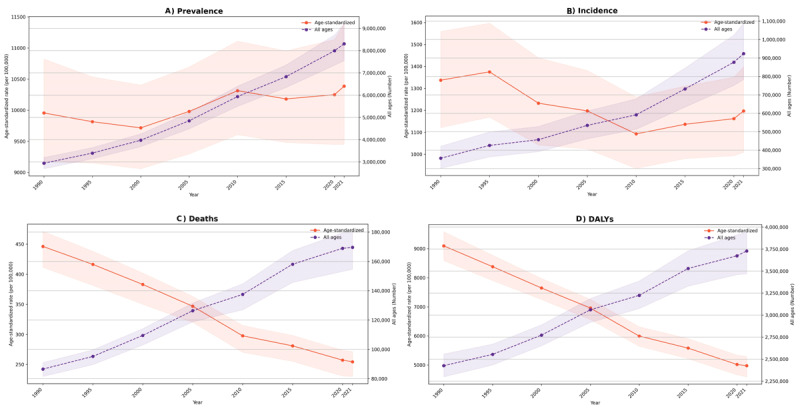
Time trend of burden measures of cardiovascular diseases (CVDs) in Iran from 1990 to 2021.

The total prevalence rose sharply, from 2.9 million individuals (95% UI: 2.7 to 3.2 million) in 1990 to 8.3 million (7.5 to 9.2 million) in 2021, reflecting a substantial increase of 182.63% (172.13 to 194.39%). Likewise, the age-standardized prevalence rate (ASPR) shifted modestly from 9,955.98 (9,174.88 to 10,821.79) to 10,385.87 (9,450.63 to 11,390.38). All age Incidence number similarly increased from 0.36 million (0.30 to 0.42 million) to 0.92 million (0.78 to 1.09 million) from 1990 to 2021, although the age-standardized incidence rate (ASIR) declined from 1,337.36 (1,121.69 to 1,559.59) to 1,197.14 (1,008.83 to 1,398.38) (Table 1, Figures 1A, and 1B).

**Table 1:** National burden of cardiovascular diseases (CVDs) in Iran in 1990 and 2021.


MEASURE	METRIC	YEAR						PERCENTAGE CHANGE		

		1990			2021					
		
		BOTH	FEMALE	MALE	BOTH	FEMALE	MALE	BOTH	FEMALE	MALE

Deaths	All ages Number	86526.77 (81550.86 to 91001.13)	38607.69 (35583.38 to 41112.91)	47919.08 (44256.07 to 51426.68)	169581.89 (154573.58 to 180461.01)	79336.39 (70863.81 to 85675.66)	90245.5 (82994.76 to 96221.33)	95.99 (83.31 to 107.56)	105.49 (91.49 to 121.0)	88.33 (71.91 to 104.18)

Deaths	Age-standardized Rate	446.27 (411.54 to 471.33)	432.79 (393.31 to 463.58)	452.27 (413.56 to 484.06)	254.52 (230.23 to 271.35)	253.68 (223.21 to 274.29)	258.76 (235.68 to 276.8)	–42.97 (–45.91 to –40.09)	–41.38 (–44.86 to –37.4)	–42.79 (–47.14 to –38.21)

DALYs (Disability-Adjusted Life Years)	All ages Number	2425354.89 (2298593.02 to 2558142.31)	1023415.86 (951893.81 to 1092198.78)	1401939.03 (1300850.55 to 1503873.77)	3727868.15 (3469264.15 to 3945805.21)	1581366.29 (1450954.14 to 1699692.15)	2146501.86 (2000574.93 to 2281116.68)	53.7 (44.83 to 62.9)	54.52 (42.8 to 66.42)	53.11 (40.16 to 65.1)

DALYs (Disability-Adjusted Life Years)	Age-standardized Rate	9095.64 (8591.46 to 9580.39)	8252.1 (7668.7 to 8776.34)	9789.6 (9037.24 to 10510.78)	4977.43 (4594.49 to 5292.4)	4455.22 (4057.54 to 4796.91)	5524.4 (5118.53 to 5882.89)	–45.28 (–48.06 to –42.42)	–46.01 (–49.33 to –42.47)	–43.57 (–47.95 to –39.28)

YLDs (Years Lived with Disability)	All ages Number	127073.37 (93356.11 to 167448.32)	59742.55 (44305.93 to 78170.59)	67330.82 (49066.37 to 89017.19)	331448.73 (236007.22 to 443324.68)	150297.95 (107863.03 to 198798.59)	181150.78 (128620.56 to 243998.79)	160.83 (147.27 to 176.28)	151.58 (136.78 to 169.1)	169.05 (155.11 to 183.85)

YLDs (Years Lived with Disability)	Age-standardized Rate	381.35 (281.07 to 500.78)	358.1 (265.75 to 463.8)	405.53 (297.69 to 536.77)	407.27 (291.48 to 540.79)	365.83 (264.52 to 481.51)	448.37 (318.29 to 598.16)	6.8 (2.36 to 11.12)	2.16 (–2.77 to 6.9)	10.56 (6.25 to 15.15)

YLLs (Years of Life Lost)	All ages Number	2298281.52 (2176983.69 to 2413175.76)	963673.31 (895810.78 to 1032014.58)	1334608.21 (1236203.33 to 1432838.05)	3396419.42 (3164494.48 to 3600600.48)	1431068.34 (1305443.53 to 1534490.9)	1965351.08 (1847758.35 to 2089599.2)	47.78 (39.03 to 57.22)	48.5 (36.47 to 61.37)	47.26 (34.33 to 59.52)

YLLs (Years of Life Lost)	Age-standardized Rate	8714.29 (8206.47 to 9172.56)	7894.0 (7283.38 to 8412.93)	9384.08 (8649.02 to 10060.15)	4570.16 (4214.83 to 4850.91)	4089.38 (3698.5 to 4395.72)	5076.03 (4746.99 to 5399.5)	–47.56 (–50.37 to –44.56)	–48.2 (–51.66 to –44.33)	–45.91 (–50.38 to –41.67)

Prevalence	All ages Number	2938840.14 (2692829.54 to 3200351.41)	1278346.83 (1172228.22 to 1385506.43)	1660493.31 (1516851.4 to 1811824.08)	8306158.04 (7542767.84 to 9207256.92)	3772277.45 (3436219.84 to 4180431.67)	4533880.59 (4102813.74 to 5042131.13)	182.63 (172.13 to 194.39)	195.09 (183.06 to 208.76)	173.04 (162.36 to 185.24)

Prevalence	Age-standardized Rate	9955.98 (9174.88 to 10821.79)	8971.07 (8248.15 to 9721.34)	10876.22 (9993.32 to 11835.32)	10385.87 (9450.63 to 11390.38)	9431.32 (8592.39 to 10342.17)	11350.32 (10307.17 to 12484.58)	4.32 (1.31 to 8.05)	5.13 (1.92 to 9.06)	4.36 (1.27 to 8.15)

Incidence	All ages Number	355773.62 (301351.93 to 420979.3)	147928.71 (127961.55 to 171702.96)	207844.91 (173625.12 to 250193.2)	923521.56 (782697.44 to 1088274.77)	402223.3 (342232.33 to 468701.65)	521298.26 (439580.4 to 620413.55)	159.58 (149.59 to 170.45)	171.9 (162.98 to 181.64)	150.81 (139.55 to 163.33)

Incidence	Age-standardized Rate	1337.36 (1121.69 to 1559.59)	1167.12 (997.32 to 1351.08)	1493.54 (1248.8 to 1754.36)	1197.14 (1008.83 to 1398.38)	1060.46 (906.87 to 1234.36)	1335.7 (1116.68 to 1574.31)	–10.48 (–12.15 to –8.77)	–9.14 (–10.81 to –7.5)	–10.57 (–12.77 to –8.34)


CVD-related deaths rose from 86,527 (81,551 to 91,001) to 169,582 (154,574 to 180,461) from 1990 to 2021. However, the age-standardized death rate (ASDR) decreased from 446.27 (411.54 to 471.33) to 254.52 (230.23 to 271.35), a reduction of 42.97% (–45.91 to –40.09). DALYs increased from 2,425,355 (2,298,593 to 2,558,142) to 3,727,868 (3,469,264 to 3,945,805), with the age-standardized DALY rate dropping significantly from 9,095.64 (8,591.46 to 9,580.39) to 4,977.43 (4,594.49 to 5,292.4) (Table 1, Figures 1C, and 1D). From 1990 to 2021, YLLs increased from 2,298,282 (2,176,984 to 2,413,176) to 3,396,419 (3,164,494 to 3,600,600), while the age-standardized YLL rate declined markedly from 8,714.29 (8,206.47 to 9,172.56) to 4,570.16 (4,214.83 to 4,850.91). YLDs showed a pronounced increase from 1990 to 2021, from 127,073 (93,356 to 167,448) to 331,449 (236,007 to 443,325), with a slight rise in the age-standardized YLD rate from 381.35 (281.07 to 500.78) to 407.27 (291.48 to 540.79) (Table 1).

#### Leading causes

**Figure 2:**
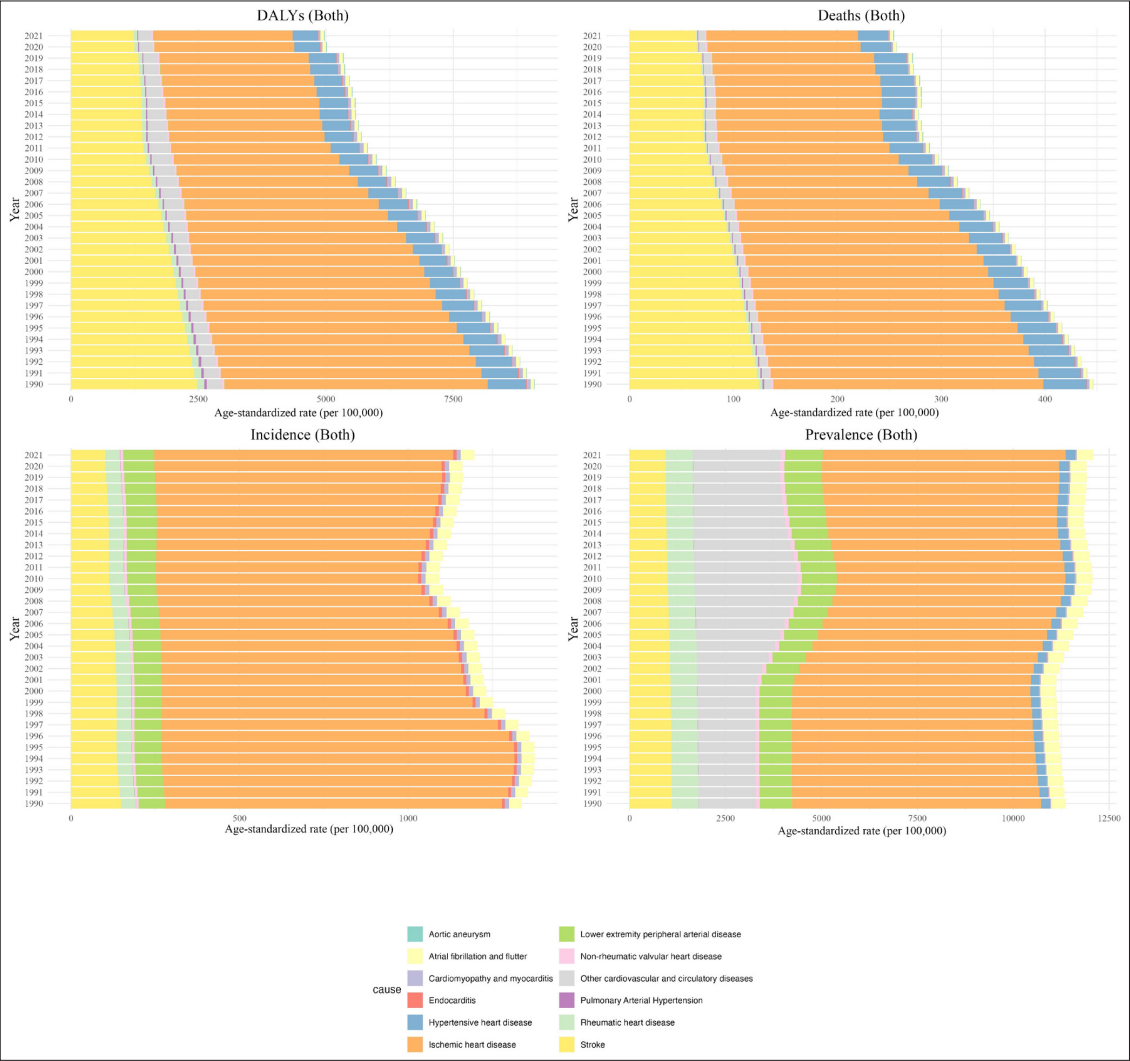
Time trend of burden measures of cardiovascular diseases (CVDs) by cause in Iran from 1990 to 2021.

Ischemic heart disease has consistently been the leading cause of CVD burden in Iran from 1990 to 2021. In 1990, the DALYs age-standardized rate (ASR) for ischemic heart disease was approximately 5166.82 (4824.54 to 5465.59), decreasing to 2731.3 (2517.27 to 2920.87) by 2021. Stroke followed a similar trend, dropping from around 2470.21 (2277.04 to 2673.16) to 1229.18 (1119.52 to 1332.6). Aortic aneurysm and lower extremity peripheral artery diseases were the only causes with a significant upward trend in DALYs ASR, while all other causes showed either a decreasing trend or no significant change. The death ASR for ischemic heart disease decreased from approximately 259.88 (238.06 to 276.23) to 146.11 (130.71 to 156.95) in 2021, with stroke deaths declining from about 125 to 65. Similar to the incidence ASR for ischemic heart disease that remained steady at approximately 6,500 and stroke around 125, ASRs of prevalence for ischemic heart disease and stroke were roughly 1,000 and 125 during the study period (Figure 2).

#### Age and sex differences

**Figure 3:**
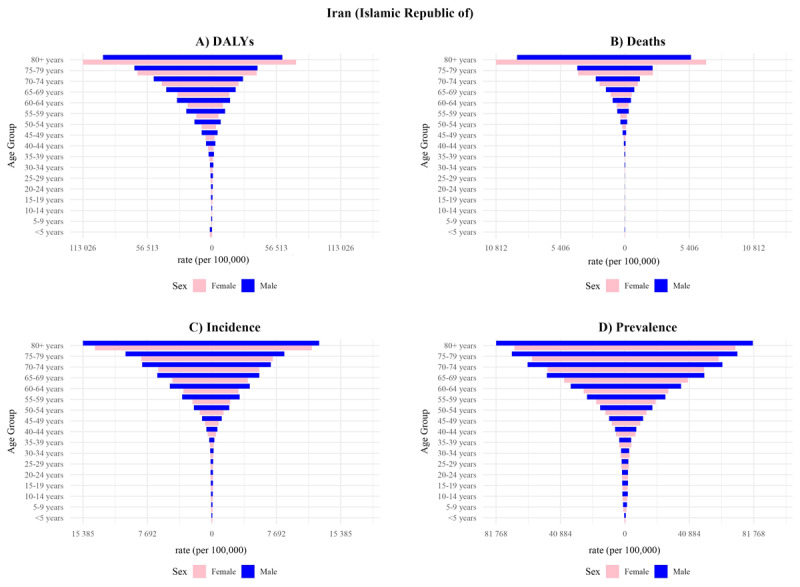
The comparison of burden measures of cardiovascular diseases (CVDs) in different age groups in Iran between 1990 and 2021.

For prevalence, females showed an increase in ASR from 8,971.07 (8,248.15 to 9,721.34) to 9,431.32 (8,592.39 to 10,342.17), reflecting a 5.13% rise. Males experienced a slightly greater increase, from 10,876.22 (9,993.32 to 11,835.32) to 11,350.32 (10,307.17 to 12,484.58). Prevalence also rose with age, peaking in the 80+ group and consistently remaining higher in males across all age categories. For incidence, females’ ASIR decreased from 1,167.12 (997.32 to 1,351.08) to 1,060.46 (906.87 to 1,234.36), while males showed a larger decline, going from 1,493.54 (1,248.80 to 1,754.36) to 1,335.70 (1,116.68 to 1,574.31). Like prevalence, ASIR rose with age, and males showed higher incidence rates across all age groups. In terms of deaths, females’ ASDR declined from 432.79 (393.31 to 463.58) 253.68 (223.21 to 274.29), Males experienced a steeper decrease, with ASDR declining from 446.27 (411.54 to 471.33) to 254.52 (230.23 to 271.35). Death rates increased with age, and while rates were initially higher in males, the sex gap narrowed in older age groups, with females exceeding males in the 75+ category. In terms of DALYs, females’ ASR dropped to 3,431.32 (3,052.39 to 3,802.55), while males showed a greater reduction and reached 4,570.16 (4,214.83 to 4,850.91). In all age groups except +80 DALYs were higher in males compared to females (Table 1, Figure 3). YLLs showed a marked ASR decrease for females, reaching 4089.38 (3698.5 to 4395.72), likewise, the ASR YLLs of males fell to 5076.03 (4746.99 to 5399.5). For YLDs, females’ ASR rose modestly to 365.83 (264.52 to 481.51) whereas males experienced a more pronounced increase and reached 448.37 (318.29 to 598.16) (Table 1).

#### Provincial trends

Examining ASPR across provinces, Ardebil displayed the highest ASPR at 12,247.46 (11,088.94 to 13,495.03), indicating a significant CVD burden. In contrast, Qom had the lowest ASPR at 9,258.99 (8,357.16 to 10,295.48), furthermore, the highest change was observed in Yazd with a 12.45% (8.09 to 17.71) increase North Khorasan led in ASIR with a rate of 1,287.47 (1,081.63 to 1,523.31), followed closely by Golestan at 1,291.81 (1,092.03 to 1,516.18), and Khuzestan at 1,314.8 (1,119.55 to 1,535.96). Tehran reported the lowest ASIR at 1,082.0 (922.58 to 1,278.82), the highest change was observed in Fars (–14.54% (–17.76 to –11.16)) (Supplementary Table 1). Golestan showed the highest ASDR (357.49 (318.3 to 388.99)), followed by West Azarbayejan (347.81 (309.11 to 392.66)), conversely Tehran had the lowest ASDR (161.53 (132.45 to 190.14)). Notably Markazi showed the highest change rate with a –52.15% (–59.55 to –43.41) decrease.

**Figure 4:**
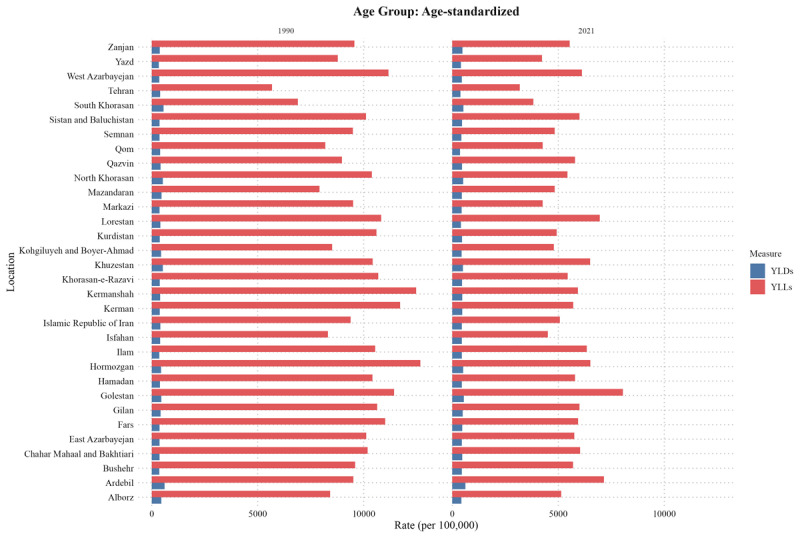
Years of life lost (YLL) and years lived with disability (YLD) comparison in Iran and its provinces between 1990 and 2021.

**Figure 5:**
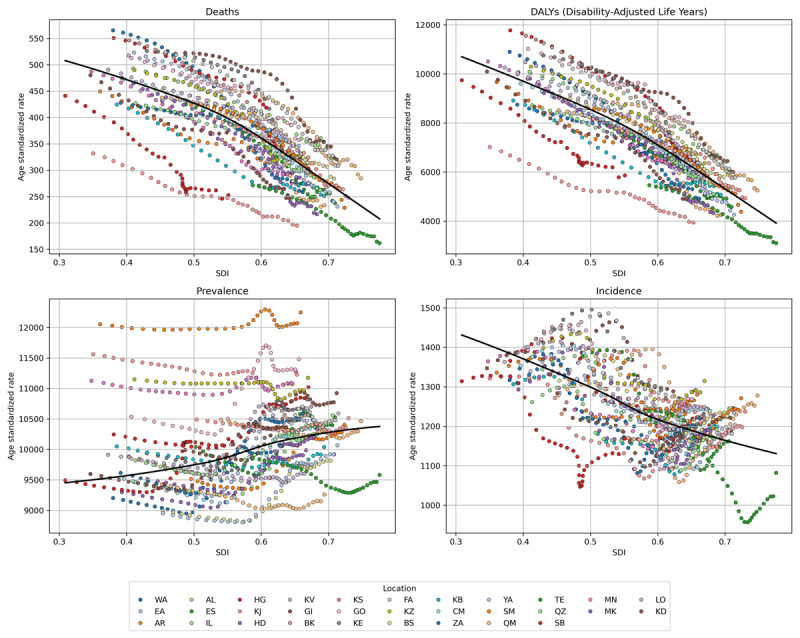
The association of burden measures of cardiovascular diseases (CVDs) with socio-demographic index (SDI) of provinces in Iran from 1990 to 2021. (Alborz: AL, Ardabil: AR, Kermanshah: BK, Bushehr: BS, Chaharmahal and Bakhtiari: CM, East Azerbaijan: EA, Isfahan: ES, Fars: FA, Gilan: GI, Golestan: GO, Hamadan: HD, Hormozgan: HG, Ilam: IL, Kohgiluyeh and Boyer-Ahmad: KB, Kurdistan: KD, Kerman: KE, South Khorasan: KJ, North Khorasan: KS, Khorasan Razavi: KV, Khuzestan: KZ, Lorestan: LO, Markazi: MK, Mazandaran: MN, Qom: QM, Qazvin: QZ, Sistan and Baluchestan: SB, Semnan: SM, Tehran: TE, West Azerbaijan: WA, Yazd: YA, Zanjan: ZA).

Golestan also experienced the highest age-standardized DALY at 7,409.36 (6,677.39 to 8,085.34), followed by Ardebil at 6,401.14 (5,719.93 to 7,041.38) and Khuzestan at 6,364.1 (5,682.52 to 7,092.16). Conversely, Tehran showed the lowest DALYs at 3100.2 (2659.43 to 3684.6), with similarly low burdens in South Khorasan at 3,944.84 (3,480.92 to 4,402.79) and Yazd at 4,259.58 (3,672.15 to 4,849.31). same as ASDR, Markazi also had the highest change rate for ASR DALY (–53.08% (–60.67 to –43.95)). Across all provinces, YLLs significantly outnumbered YLDs, contributing more to the overall DALYs (Figure 4). While a higher SDI was associated with lower deaths, DALYs, and incidence of CVDs in Iran, it was negatively associated with prevalence (Figure 5).

#### Decomposition analysis

Decomposition analysis indicated that of the 159.6% increase in CVD incidence rates in Iran, 49.5% and 136.2% were attributed to population growth and changes in age structure, respectively, while changes in incidence rates contributed negatively (–26.1%). A similar pattern was observed in both males and females (Table 2). Decomposition analysis showed an increase in incidence across all provinces in both sexes; moreover, in all provinces incidence rate change had a negative effect on incidence while population age structure and population growth had a positive contribution to incidence (Supplementary Table 2). For ASDR, Golestan showed the highest rate at 357.49 (318.3 to 388.99), followed by West Azarbayejan 347.81 (309.11 to 392.66), and Ardebil at 322.88 (286.55 to 356.66). Tehran had the lowest ASDR at 161.53 (132.45 to 190.14), with low rates also in South Khorasan at 194.76 (166.99 to 218.65) and Markazi at 216.53 (184.37 to 245.65) (Supplementary Table 1).

**Table 2:** Decomposition analysis of cardiovascular diseases (CVDs) incidence by sex between 1990 to 2021 in Iran.


SEX	NEW CASES		EXPECTED NEW CASES		CONTRIBUTION OF EACH FACTOR			OVERALL CHANGE (%)
		
	1990	2021	POPULATION GROWTH	POPULATION GROWTH + AGING	POPULATION GROWTH (%)	AGE STRUCTURE CHANGE (%)	INCIDENCE RATE CHANGE (%)	

Both	355,774	923,522	531,827	1,016,378	49.5	136.2	–26.1	159.6

Female	147,929	402,223	221,770	440,182	49.9	147.6	–25.6	171.9

Male	207,845	521,298	309,832	576,195	49.1	128.2	–26.5	150.8


#### Risk Factors

**Figure 6:**
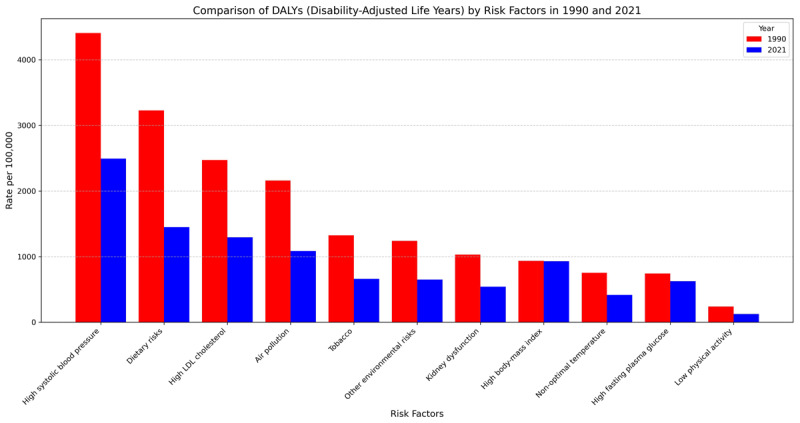
Comparison of disability-adjusted life years (DALYs) attributable to each risk factor in Iran between 1990 and 2021.

The age-standardized DALYs attributed to risk factors for cardiovascular diseases in Iran indicated that high systolic blood pressure was the leading contributor at the national level in both 1990 and 2021, with its impact remaining consistently significant and surpassing other risk factors across all provinces. High LDL cholesterol and dietary risks were the next most prominent contributors to DALYs nationwide (Figure 6).

## Discussion

### Overview

This study highlights three critical trends in Iran’s cardiovascular disease (CVD) burden: 1) a 182.63% rise in CVD prevalence (8.3 million cases in 2021) driven by population aging, despite declining ASRs; 2) noticeable provincial disparities, with low-SDI provinces experiencing higher mortality/DALYs and high-SDI provinces reporting greater incidence/prevalence; and 3) modifiable risk factors—high systolic blood pressure, dietary risks, and high LDL cholesterol—remaining the largest contributors to CVD burden. These findings highlight the urgency of tailored, equity-focused interventions for Iran’s aging population and socioeconomically diverse provinces.

#### Subnational trends: Addressing inequities

Provinces with high SDI (e.g., Tehran, Isfahan) exhibited lower mortality but higher CVD prevalence, likely reflecting improved healthcare access and diagnostic capacity In contrast, low-SDI provinces face elevated mortality due to limited access to cardiac surgeries (19,20,21,22). essential medications (23) and adherence challenges. These disparities mirror global patterns where socioeconomic inequities amplify CVD outcomes. Prioritizing healthcare infrastructure (e.g., cardiac care units, medication supply chains) in underserved regions could mitigate mortality gaps.

#### Age and sex disparity

CVD burden disproportionately affects older adults in Iran, contrasting with patterns in many LMICs where younger populations bear a larger burden. This aligns with physiological aging processes (arterial stiffness, chronic inflammation) (24,25). and Iran’s demographic transition. Notably, males had higher ASRs across most age groups, likely due to greater tobacco use, abdominal obesity, and lower HDL cholesterol (26). While CVDs in many low- and middle-income countries tend to primarily affect younger, working-age populations, Iran presents a different pattern, with a more significant burden in older age groups. This is partly due to the country’s aging population, where the risk of cardiovascular disease is compounded by the rise in chronic conditions. Moreover, both prevalence and mortality rates from CVD are consistently higher in males across various age groups except the +75 years age group, a pattern that may stem from multiple biological, behavioral, and social factors. Men tend to have higher rates of certain risk factors, such as tobacco use, and abdominal obesity, and also generally have lower levels of HDL cholesterol, increasing their CVD risk (27,28). However, after menopause women are at more risk for CVDs (29). These is because premenopausal women have the protective benefits of estrogen which males and post-menopausal women do not (30,31).

#### Risk factors and policy implications

In a study on burden of CVDs in USA, Ford et al. estimated that 47% of the observed decline in coronary heart disease (CHD) mortality was as a result of medical and surgical treatments, while 44% was caused by reductions in major risk factors (30). Studies on primary prevention, such as one by O’Flaherty, indicate that substantial dietary improvements could prevent up to 30,000 cardiovascular deaths in the UK between 2006 and 2015, compared to just 12,500 fewer deaths if current trends continued (31). Similar findings have been reported in studies analyzing European (32) and American (33) data. In Iran, where dietary risks remain the leading contributor to CVD burden, policy action must expand beyond existing efforts. These findings are comparable to Iran since the risk factor with the most burden was dietary risk in the country. To address this, policymakers should prioritize stricter regulations on ultra-processed foods, including mandatory front-of-package warning labels and taxes on high-sodium and high-sugar products, alongside subsidies for healthy foods like fruits and whole grains. Until now, the country has implemented an NCDs roadmap aligned with WHO best-buy recommendations, which includes policies aimed at reducing sugar, fat, and salt intake (32). Over the past decade, studies have reported significant improvements in blood lipid profiles within the Iranian population. This progress can be attributed to efforts such as reducing Trans Fatty Acids (TFA) in the oil industry and raising public awareness about the adverse health effects of TFA consumption (33). As a result, levels of total cholesterol and non-HDL cholesterol have decreased, while HDL cholesterol levels have increased (34). High BMI and HTN rank among the leading contributors to Iran’s CVD burden. Despite national efforts to develop nutrition-oriented policies and integrate dietary strategies into development programs, obesity rates remain persistently high due to a lack of targeted, actionable interventions (35,36). For instance, while initiatives to reduce salt and trans-fatty acids have shown success (32,33) dedicated policies to curb obesity—such as regulating ultra-processed foods or promoting physical activity—are notably absent. This gap underscores the need for a holistic approach involving multisectoral collaboration (e.g., food industry regulations, urban planning, public education) to address obesity’s root causes. Compounding this issue is the rise in physical inactivity, as evidenced by the STEPS study, which reported increased sedentary behavior in Iran between 2016 and 2021 (25). Among the various challenges, barriers to physical activity among the elderly in Iran and globally can be categorized into three main areas: interpersonal factors, such as lack of a companion, absence of professional guidance, family responsibilities, and social pressures; intrapersonal factors, including physical limitations, time constraints, lack of interest, laziness, financial cost, security concerns, and fear of falling; and environmental factors, such as traffic, weather conditions, and physical barriers to walking (26). To combat this, multisectoral collaboration is critical—for example, enforcing urban planning policies that create walkable communities and green spaces, specially in high-risk provinces like Golestan and Ardebil, and launching nationwide campaigns to reduce sedentary behavior. Moreover, subsidizing gym memberships for low-income populations and integrating physical activity programs into primary healthcare services could be beneficial.

For HTN, it is worth emphasizing that despite breakthroughs in technology, treatment, care, and disease prevention, the prevalence of HTN has not reduced over time in Iran (37,38). This highlights the need for more effective preventive and treatment-focused strategies. Policy-makers should adopt province-specific strategies: expanding access to affordable antihypertensive medications in low-SDI provinces and enhancing community-based screening programs in high-SDI provinces like. Campaigns to raise public awareness of the dangers of hypertension and the significance healthy lifestyle can be effective (39). Stricter regulations on food labeling, especially regarding sodium content, and initiatives to reduce the consumption of unhealthy foods could further help decrease hypertension rates (40).

#### Limitations and strengths

Despite many strengths of this study, several limitations should be noted. GBD project provided the data used in this analysis, which may not fully represent regional variations in health determinants or healthcare quality. Additionally, this study does not account for other socioeconomic determinants of health, such as income and education, which may significantly impact CVD outcomes. These societal aspects should be investigated in future studies to give a more thorough picture of Iran’s CVD burden. Additionally, longitudinal studies would be valuable for evaluating how risk variables and health outcomes vary over time. Notwithstanding these drawbacks, this study has a number of advantages, such as a broad timeframe and the application of age-standardized rates, which enable insightful comparisons across time and between geographical areas. Additionally, the provincial-level data offer insightful information on regional inequities, enabling focused health initiatives. These understandings are essential for creating plans that take into account the particular requirements of different groups and geographical areas, which will ultimately result in public health programs that are more successful.

## Conclusion

The CVD prevalence has increased by 182.6% in prevalence since 1990, driven by population aging and growth, though ASR mortality and DALY rates have declined due to healthcare advancements. Notable provincial disparities persist: low-SDI provinces like Golestan and Ardebil face higher mortality and DALYs linked to limited healthcare access, while high-SDI provinces such as Tehran reported higher prevalence, as a consequence of better diagnostics and aging populations. IHD and stroke remain leading causes, with aortic aneurysm emerging as a growing concern, highlighting the necessity of addressing modifiable risks like HTN, poor diet, and physical inactivity. Despite Iran’s progress in reducing trans fats and improving lipid profiles through policies, gaps persist in tackling obesity and sedentary lifestyles, particularly among males, who exhibit higher CVD burden except in older age groups where female risk escalates post-menopause. Equitable resource allocation to improve primary care in underserved provinces, stricter food industry regulations, and sex-specific interventions targeting smoking and metabolic risks are critical. Prioritizing multisectoral strategies—integrating geriatric care, public health campaigns, and infrastructure for physical activity—can attenuate CVD’s toll, leveraging Iran’s demographic transition and fostering collaboration between government, healthcare, and communities to safeguard future generations.

## Data Accessibility Statement

All data are available from: https://github.com/mahsaf2000/CVD_GBD_supplement.
